# Phosphoprofile reorganization of the actin binding protein Drebrin during long term depression

**DOI:** 10.3389/fnmol.2025.1697642

**Published:** 2025-11-05

**Authors:** Rafaela Pedro Silva, Till G. A. Mack, Marieluise Kirchner, Philipp Mertins, Britta J. Eickholt, Patricia Kreis

**Affiliations:** ^1^Institute of Biochemistry and Molecular Biology, Charité - Universitätsmedizin Berlin, Berlin, Germany; ^2^Department of Biology, Chemistry and Pharmacy, Freie Universität Berlin, Berlin, Germany; ^3^Core Unit Proteomics, Berlin Institute of Health (BIH) at Charité and MDC, Berlin, Germany

**Keywords:** Drebrin, actin, synaptic plasticity, long-term depression, phosphorylation, calpain

## Abstract

Drebrin (DBN), an actin-binding protein critical for the structural integrity and function of dendritic spines, is highly phosphorylated at steady state in neurons. Here, we investigate the phosphorylation dynamics of DBN in the context of chemically induced long-term depression (cLTD), a synaptic plasticity model mimicking activity-dependent weakening of synapses. Using biochemical analyses and mass spectrometry analyses, we show that DBN undergoes rapid and robust changes in phosphorylation following cLTD induction. Notably, cLTD triggers a marked decrease in many DBN phosphorylation sites, accompanied by proteolytic cleavage of the protein, suggesting a tightly regulated mechanism linking post-translational modification to structural remodelling of the synapse. Our findings highlight the dynamic regulation of DBN by phosphorylation during synaptic depression and support its potential role as a modulator of activity-dependent synaptic plasticity.

## Introduction

Drebrin (DBN) regulates cytoskeletal functions during neuronal development, and is thought to contribute to structural and functional synaptic changes associated with ageing and Alzheimer’s disease (AD) (Kreis et al., 2019; Shirao et al., 2017; Sonego et al., 2025). It is a conserved actin-binding protein (ABP), which binds and stabilizes filamentous actin (F-actin), decreasing actin filament turnover (Mikati et al., 2013; Sharma et al., 2011). DBN exists as two major isoforms, DBN E and DBN A, generated through alternative splicing; DBN A is characterized by the presence of an additional 45-amino acid insert not found in DBN E. Originally identified in the brain, DBN E represents the embryonic isoform predominantly expressed during early neuronal development, while DBN A is the adult isoform, expressed specifically during the formation of synaptic connections (Shirao et al., 2017). In fact, DBN E is also expressed at a lower level in other cells of the brain such as astrocytes, and in further tissues including the stomach, kidneys and testis (Keon et al., 2000).

The core DBN domain structure consists of an ADF/Cofilin homology domain, two actin-binding domains, the helical and coiled-coil regions, (Worth et al., 2013), which are followed by a small proline-rich region and two Homer-binding motifs (Shirao and Sekino, 2017). In this protein, the majority of functional domains are located within the N-terminal region, while the C-terminal portion has been characterized as intrinsically disordered (Varga et al., 2025). DBN not only binds to F-actin, it is also known to interact with other cytoskeletal proteins including EB3, and post-synaptic architectural proteins such as Homer and Spikar (Geraldo et al., 2008; Yamazaki and Shirao, 2017). To date, very little data is available on the functional difference between DBN E and DBN A. Recently, Srapyan and colleagues showed that compared to DBN E, DBN A has a stronger actin capping activity, leading to a decrease in DBN A-bound actin polymerisation and a decrease in DBN A-bound actin severing by cofilin (Srapyan et al., 2025).

DBN A is highly enriched in dendritic spines, where it plays a critical role in regulating spine morphology and dynamics (Biou et al., 2008; Ivanov et al., 2009). As a key component of the spine’s structural framework, DBN A can translocate in and out of spines in response to synaptic activity and long-term potentiation, thereby contributing to both structural and functional synaptic plasticity (Bosch et al., 2014; Sekino et al., 2006). Overexpression of DBN interferes with the structural organisation within dendritic spines, leading to an accumulation of actin and the formation of long protrusions (Mizui et al., 2005). Interestingly, DBN knockout mice (Dbn^–/–^) appear largely normal, exhibiting no obvious defects in brain morphology or neuronal connectivity. Neurons from these mice display normal morphology, neurite outgrowth, and basal synaptic transmission, suggesting that the absence of DBN alone is not sufficient to cause synaptic dysfunction (Willmes et al., 2017). Subsequent studies revealed that oxidative stress or epileptic activity can unmask the effects of DBN loss, leading to impairments in synapse integrity and network activity (Klemz et al., 2021; Kreis et al., 2019). Mechanistically, DBN was shown to integrate cytosol-induced stress signalling pathways that regulate the actin cytoskeleton, thereby providing essential protection of synapses from local stress responses (Kreis et al., 2019).

In line with a protective effect of DBN, it has been reported that brains of patients with cognitive impairment associated with aging show a progressive loss of DBN, with symptom severity correlating with the extent of DBN reduction, suggesting that DBN may safeguard against age-induced dendritic spine degeneration (Counts et al., 2012; Harigaya et al., 1996; Hatanpää et al., 1999; Shim and Lubec, 2002). The mechanisms regulating DBN at the synapse, particularly during synaptic decline, still remain poorly understood. To address this gap, we investigated post-translational modifications of DBN in the context of long-term depression (LTD) in an unbiased manner. LTD is classically characterized by a sustained reduction in synaptic efficacy, which, over time, leads to the shrinkage or loss of dendritic spines and is associated with impairments in higher cognitive functions (e.g., memory loss) (Citri and Malenka, 2008; Lüscher and Malenka, 2012).

Here, using mass spectrometry to assess the phosphorylation status of DBN during a chemically induced long-term depression (cLTD) protocol, we reveal dynamic changes in DBN phosphorylation. We identify specific phosphorylation sites that are either upregulated or downregulated, as well as a cleavage event that coincides with these changes. Overall, our findings suggest that DBN undergoes tightly regulated post-translational modifications during LTD, potentially contributing to synaptic remodelling and plasticity under conditions of synaptic weakening.

## Materials and methods

### Mouse primary cultures

Cortices were dissected from E16.5 C57BL/6 mice embryos and were dissociated using 20 U/mL papain (LS003126, Worthington Biochemical) in Hank’s balanced salt solution (HBSS, 14170070 Thermo Fisher) for 30 min at 37 °C. Enzymatic digestion was stopped using an enzyme inactivating solution containing albumin (2.5 mg/mL, A2153 Sigma) and trypsin inhibitor (2.5 mg/mL, T9253 Sigma) in DMEM complete medium (31966 Gibco). A washing step using growth medium (Neurobasal medium supplemented with 10% fetal bovine serum (FBS, P30-3031 PAN Biotech), 2% B27 (17504044 Thermo Fisher), 1% Glutamax (11574466 Fisher Scientific) and 1% Penicillin/Streptomycin (15140122 Thermo Fisher) followed. Tissue was then triturated in the same medium using P200 pipette tips until a dense single-cell suspension was obtained.

For biochemical and mass spectrometry analysis, single-cell suspension cortical neurons were plated at a density of 1Mio/well in 6-well plates previously coated with 15 μg/m, poly-DL-ornithine (P8638 Sigma) resuspended in borate buffer (50 mM boric acid, 12.4 mM sodium-tetraborate) to avoid astrocytic growth. For cellular imaging, the same single-cell suspension was plated on a 12-well plate at a density of 150 k/well on glass coverslips (Ø18 mm, HKH7.1 Roth) previously washed with absolute ethanol (>99%, 5054.3 Roth), and coated with 30 μg/mL poly-DL-ornithine (P8638 Sigma) followed by 30 μg/mL laminin (L2020 Sigma).

Two hours after plating, the growth medium was removed and replenished by maintenance medium (Neurobasal medium supplemented with 2% B27, 0.5% Glutamax and 1% Penicillin/Streptomycin). Neurons were maintained in a humidified atmosphere at 37 °C and 5% CO_2_ for the remaining experimental time.

### Chemical long-term depression

cLTD was evoked based on previous work (Kallergi et al., 2022; Kapitein et al., 2011; Martínez-Mármol et al., 2016). In brief, DIV15-16 neurons were gently washed twice using a Mg^2+^-free phosphate buffered solution (PBS, 9124.1 Roth) supplemented with 2 mM Ca^2+^ to ensure both N-methyl-D-aspartate (NMDA) receptor availability and baseline neuronal function, respectively. NMDA receptor agonist NMDA (454575-100MG Merck) was then incubated at 50 μM for 5 min in the previously mentioned PBS solution in a 37 °C, 5% CO_2_ atmosphere. After incubation, the solution containing the drug was promptly removed and neurons were again incubated with maintenance medium for the remaining 2 h of the experimental protocol before lysis or fixation.

### Protein lysate collection and quantification

Two hours after evoking cLTD, whole cells were homogenized in 2% SDS, 150mM NaCl and 50 mM TRIS buffer supplemented with protease (Calbiochem set III, 539134-1 Merck) and phosphatase (1 mM Na_2_MO_4_, 1 mM NaF, 20 mM β-glycerophosphate, 1 mM Na_3_VO_4_, 500 nM cantharidin) inhibitors using a cell scraper. The lysate was collected without further centrifugation steps and snap frozen in liquid nitrogen. Smaller aliquots of the lysate were used for protein quantification analysis using BCA Thermo Scientific Pierce Protein Assay (23225 Thermo Scientific). Protein lysates for further in-house western blotting assays were then prepared with 4 × Roti-Load (K929.2 Roth).

### Immunocytochemistry

Neuronal cultures assessed for overall integrity were fixed in 4% paraformaldehyde (PFA, 28794.295 VWR International) supplemented with 4% sucrose in a cytoskeleton-stabilizing PHEM buffer (PIPES, HEPES, EDTA and MgCl_2_, pH 7.4) for 20 min at DIV15-16. Neurons were permeabilized in PHEM with 0.2% Triton-X100 for 10min and blocked in PHEM with 4% goat serum for 1 h. Primary antibody MAP2 was incubated in 4% goat serum overnight at 4 °C. After three washes with room temperature PHEM, secondary antibody Alexa647 was incubated in PHEM for 1h. After secondary incubation, three washes using PHEM with Hoechst 33342 (ICT-639 Antibodies Inc.) were performed before mounting using Prolong Gold antifade (P36930 Invitrogen).

### Western blotting

On average, 5–15 μg of protein was loaded on an SDS-polyacrylamide gel electrophoresis (SDS-PAGE) gel. Western blot analysis was performed with minor modifications to previously described (Schrötter et al., 2016). Protein lysates were loaded on 15% SDS gels, stacked at 80V for 30 min, and separated at 120V for roughly 60 min. Proteins were transferred to a nitrocellulose membrane using a wet blot tank system (Bio-Rad) for 2 h. The membranes were then blocked for 1 h at room temperature with 5% milk before incubating with the primary antibodies.

Primary antibodies were prepared in 5% milk and incubated overnight at 4 °C. Following primary incubation, membranes were washed four times with TBS-T at room temperature for 5 min. Secondary antibodies were also prepared in 5% milk, and membranes were then incubated for 1 h at room temperature. Following secondary incubation, membranes were washed four times with TBS-T before being developed with ECL (W1001 Promega) using the Fusion SL system from Vilber Lourmat.

### Antibodies

**Table:** 

Antibody	Use	Catalogue number	Company
Anti-mouse Alexa647	ICC 1:1000	715-605-150	Dianova
Anti-mouse HRP	WB 1:10 000	PI-2000	Vector Laboratories
Anti-rabbit HRP	WB 1:3000	PI-1000	Vector Laboratories
Mouse anti-α-tubulin	WB 1:10 000	T9026	Merck
Mouse anti-MAP2	ICC 1:500	M9942	Sigma
Rabbit anti-DBN (epitope 304-354)	WB 1:1000	DF12388	Affinity Biosciences
Rabbit anti-DBN (epitope 143-160)	WB 1:1000	M05530	Boster Bio

### Mass spectrometry based phosphoproteomics

Lysates (in 2% SDS, 150 mM NaCl, 10mM dithiothreitol, 40 mM 2-chloroacetamide, 50 mM TRIS-HCl (pH 8.5), with phosphatase and protease inhibitors) were heated for 10 min at 95 °C, cooled down to room temperature and incubated with Benzonase (50 units) for 30 min at 37 °C. After quenching with 80 mM dithiothreitol, samples were centrifuged for 10 min at 12,000 *g* and the soluble fraction was subjected to SP3 clean-up and tryptic digest as previously described (Hughes et al., 2019). Briefly, 200 μg protein aliquots were diluted in acetonitrile (70% final concentration), and paramagnetic beads containing 1:1 hydrophilic and hydrophobic beads were added at a protein/bead ratio of 1:10. After 20 min incubation on a rotor wheel, samples were washed twice with 70% ethanol and once with 100% acetonitrile. Digestion buffer (50 mM ammonium bicarbonate, 4 μg trypsin and LysC) was added and samples were incubated over night at 37 °C. The peptide containing supernatant was collected, acidified with formic acid (1% final concentration) and dried down. Samples were resolved in 50 mM HEPES (pH 8) and labelled with 16-plex tandem mass tag (TMTpro, Fisher Scientific) reagents following the vendors instructions. After combining all samples and C18 SepPak-based clean-up (Waters, 200 mg/1cc), samples were fractionated by high-pH reversed phase off-line chromatography (1290 Infinity, Agilent) and pooled into 30 fractions. 10% of each fraction was taken out for global proteome measurements. The remaining 90% were further pooled onto 15 fractions and applied to IMAC based phosphopeptide enrichment using Fe(III)-IMAC cartridges and the AssayMAP Bravo Platform (Agilent Technologies). For LC-MS/MS measurements, peptides were reconstituted in 3% acetonitrile with 0.1% formic acid and separated on a reversed-phase column [20 cm fritless silica microcolumns with an inner diameter of 75 μm, packed with ReproSil-Pur C18-AQ 1.9 μm resin (Dr. Maisch GmbH)] using a 98 min gradient with a 250 nl/min flow rate of increasing Buffer B (90% ACN, 0.1% FA) concentration (from 2 to 60%) on a High Performance Liquid Chromatography (HPLC) system (Thermo Fisher Scientific) and analyzed on a Q Exactive HF-X instrument (Thermo Fisher Scientific). The mass spectrometer was operated in data-dependent acquisition mode using the following settings: full-scan automatic gain control (AGC) target 3E6 at 60K resolution; scan range 350–1500 m/z; maximum injection time 10 ms; MS/MS scan AGC target of 1E5 at 45K resolution; maximum injection time 86 ms (global) and 120 ms (phosphopeptide enriched samples); normalized collision energy of 30 and dynamic exclusion time of 30 s; precursor charge state 2–6, 20 MS2 scans per full scan. RAW data were analyzed with MaxQuant software package (v 1.6.10.43) using the Uniprot databases for mouse (2022–03). The search included variable modifications of methionine oxidation, N-terminal acetylation, deamidation (N and Q) and phosphorylation (STY) and fixed modification of carbamidomethylated cysteine. Reporter ion MS2 for TMT16 was selected (internal and N-terminal) and TMT batch specific corrections factors were specified. The FDR (false discovery rate) was set to 1% for peptide and protein identifications. Unique and razor peptides were included for quantification.

The resulting text files were used for data analyses. Reverse hits, potential contaminants and proteins only identified by site were excluded. Protein groups and phosphosite tables were further filtered for 100% valid value. Corrected reporter ion intensities were log2 transformed and normalized using median z-score within each sample. Proteomics data is available as Supplementary Data. The nomenclature used throughout this work refers to the Human DBN A isoform.

### Statistical analysis and representations

Statistical analysis and representations were performed using GraphPad Prism version 9 (GraphPad Software, California, USA). Statistical significance was evaluated with unpaired *t*-test two-stage step-up (Benjamini, Krieger and Yekutieli) and statistically significant values were defined by a *p*-value of less than 0.05.

## Results

### Drebrin is a phosphoprotein

**FIGURE 1:**
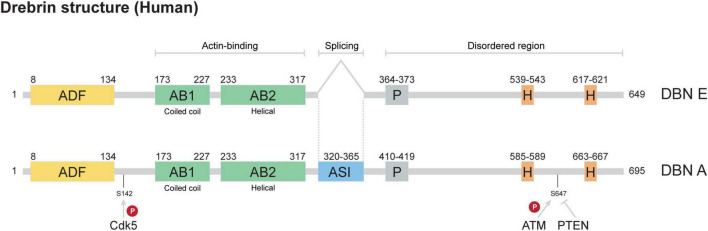
DBN structure. The core structure of DBN consists of four main domains: ADF (ADF/cofilin homology domain), AB1 and AB2 (actin-binding domains 1 [coiled-coil] and 2 [helical]), P (proline-rich motif), and H (Homer-binding motifs). The DBN A isoform contains an additional stretch of amino acids enriched in serines, generated through alternative splicing (referred to as ASI; adult-specific insert). The figure highlights the actin-binding regions, the alternatively spliced segment, and intrinsically disordered regions. Also indicated are two characterized phosphorylation sites (S142 and S647) together with their regulatory kinases/phosphatases (Cdk5, ATM/PTEN).

DBN is a highly phosphorylated protein, with 40 phosphorylation sites identified through shotgun phosphoproteomic analyses in accordance with the latest information on phosphorylation databases such as PhosphositePlus, PhosphoELM and qPTM. These sites have been detected across various cancer types, including breast, lung, prostate, and ovarian cancers (e.g., pS5, pS7, pS141), as well as in T-cell leukemia and B-cell lymphomas (pS142). In the nervous system, approximately 20 phosphosites have been reported from studies using cell lines, primary cortical cultures, synaptoneurosomes, and brain tissue. Despite the large number of sites identified, only two—S142 and S647—have been functionally characterized: phosphorylation of S142 by Cdk5 regulates DBN’s conformational state (open/closed), while phosphorylation of S647 by ATM Kinase or dephosphorylation by PTEN influences its protein stability (Figure 1; Kreis et al., 2013, 2019; Worth et al., 2013).

### Drebrin is cleaved during chemically induced long-term depression

**FIGURE 2:**
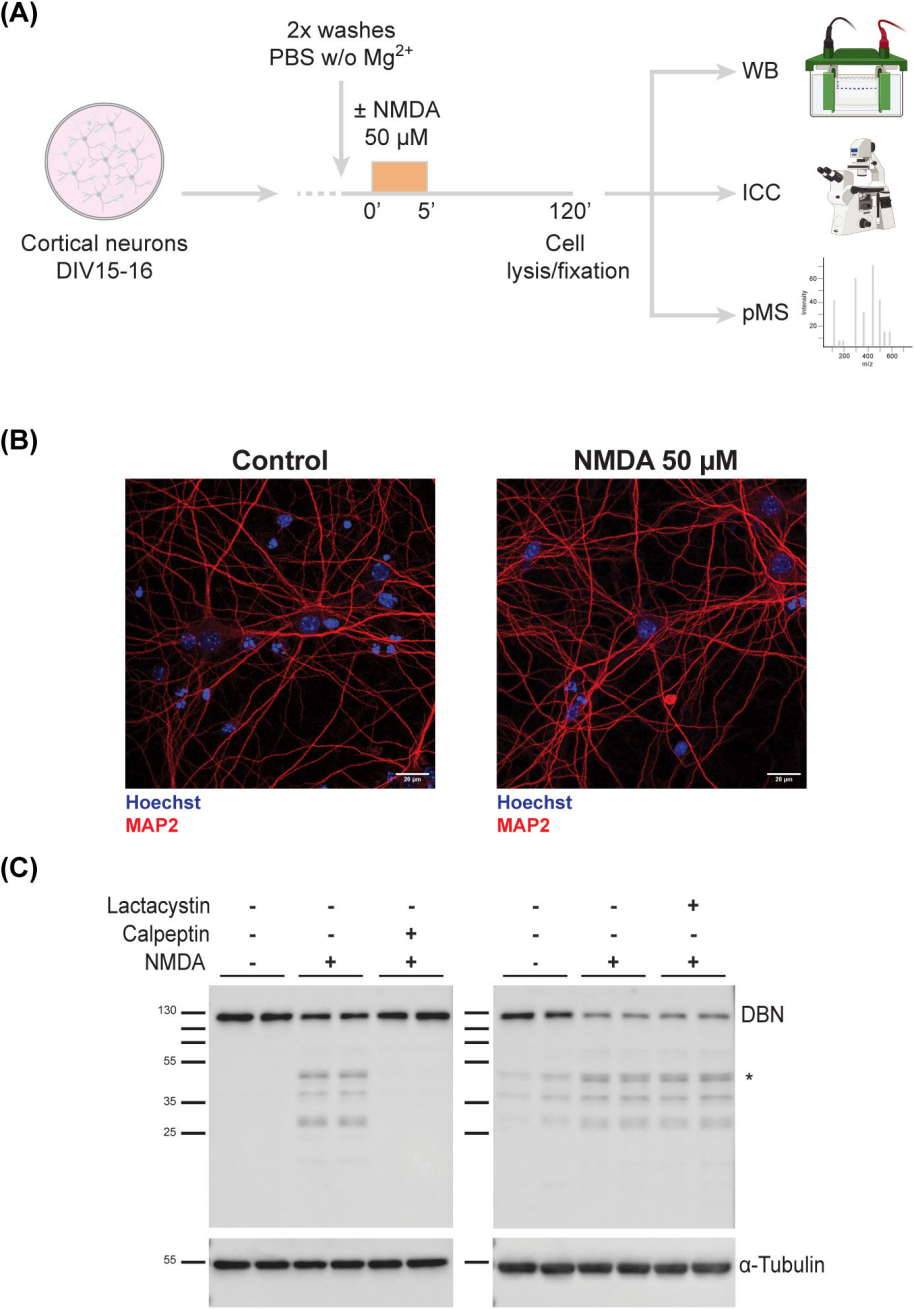
Full-length DBN is cleaved in response to synaptic weakening. **(A)** Work flow of cLTD on *in vitro* cultured cortical neurons **(B)** DIV15-16 cortical neurons were immunolabelled to assess viability using MAP2 (dendritic tree marker in red) and Hoechst (nuclear staining in blue). Treated neurons show no signs of neurodegeneration. Scale bar: 20 μM. **(C)** Upon NMDA treatment, DBN is cleaved into several sized fragments between 25 and 50 kDa, with the most prominent one around 45–50 kDa (*). This phenotype is only reversible upon the co-application of calpeptin, a calpain inhibitor (left membrane), but not lactacystin, a proteasomal inhibitor (right membrane). α-Tubulin was used as a loading marker. Molecular weight protein ladder is in kilodaltons.

Given the limited functional evidence for most DBN phosphosites, we undertook a comprehensive phosphoproteomic analysis to investigate if and how phosphorylation of DBN is regulated at the synapse. To model synaptic depression, we employed a well-established cLTD paradigm. Cortical neurons at DIV15–16 were treated with NMDA for 5 min and analyzed 120 min post-stimulation (Figure 2A), following previously described protocols (Kallergi et al., 2022; Kapitein et al., 2011; Martínez-Mármol et al., 2016; Supplementary Figure 1).

To confirm neuronal integrity post-treatment, cultures were immunolabeled with MAP2, a dendritic microtubule marker. MAP2 staining showed that NMDA-treated neurons remained viable and retained an intact dendritic cytoskeleton, indicating that the brief NMDA exposure did not compromise neuronal health (Figure 2B).

We next examined the effect of NMDA treatment on DBN protein abundance using western blotting. NMDA stimulation led to a marked reduction in full-length DBN levels and the emergence of multiple low-molecular-weight fragments, with a prominent fragment detected at approximately 45–50 kDa. To determine the mechanism underlying DBN degradation—whether via calpain-mediated proteolysis or the ubiquitin-proteasome pathway—we co-treated neurons with NMDA and either calpeptin (a calpain inhibitor) or lactacystin (a proteasome inhibitor). Only calpeptin effectively prevented the NMDA-induced reduction of full-length DBN (Figure 2C). These findings indicate that DBN cleavage during cLTD is mediated by calpain, consistent with previous reports of calpain-dependent DBN degradation under excitotoxic conditions (Chimura et al., 2015). Western blot analysis and *in silico* calpain cleavage site prediction suggests that the cleavage product localizes to the N-terminal region of DBN (Supplementary Figure 2). The exact cleavage site and full sequence of the fragment still remain to be identified.

### Drebrin is dynamically regulated

**FIGURE 3:**
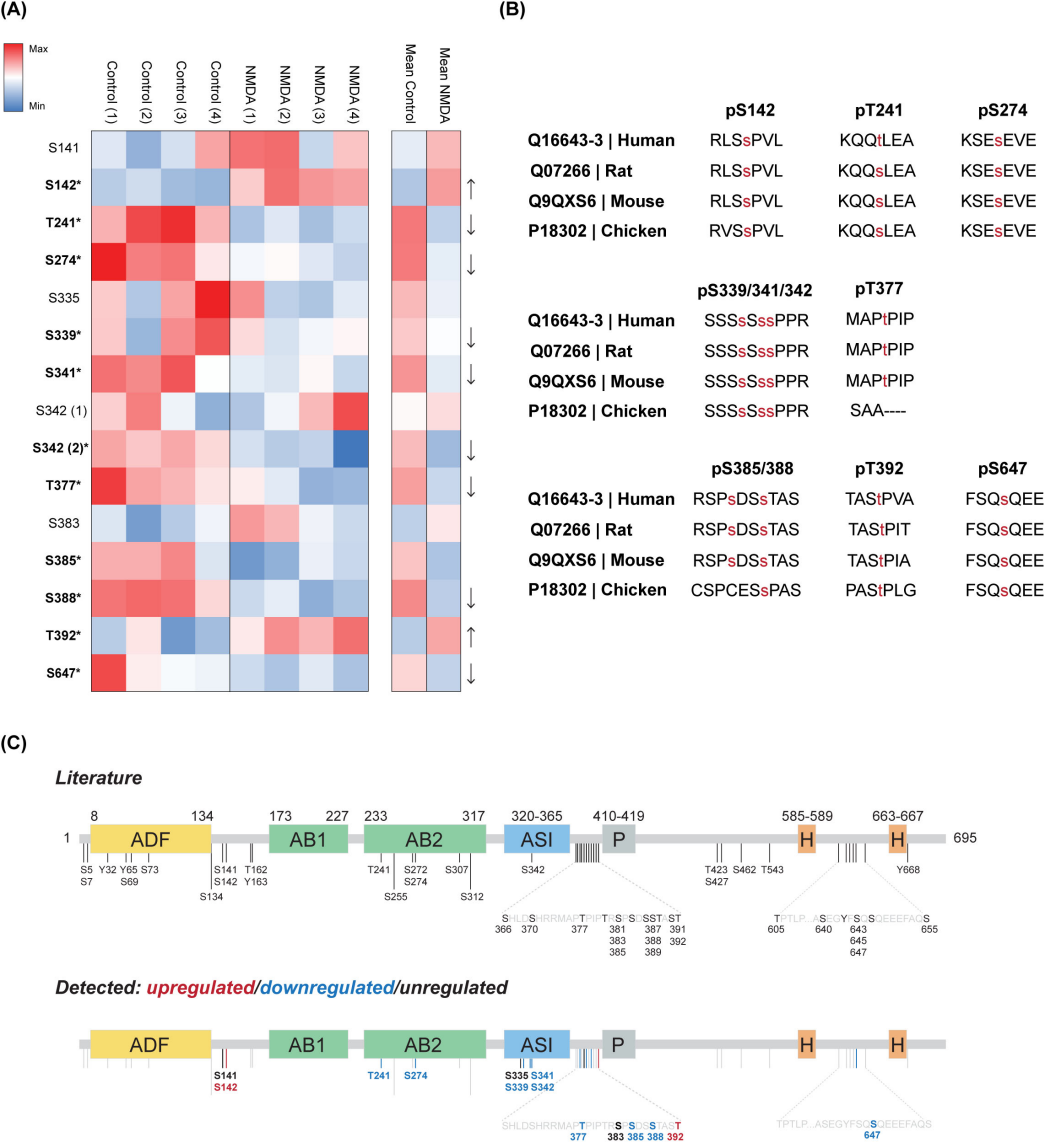
DBN phosphorylation is altered in response to cLTD. **(A)** Heatmap shows the ratio of occupation at each DBN site by phosphorylation identified during proteomics, in four individual neuronal cultures (named 1–4). Values represented are the log_2_ intensity values normalized to the median z-score within each sample. Increasing red or blue values represent higher levels of phosphorylation or lower levels of phosphorylation, respectively. Significantly altered phosphorylation levels are indicated in bold together with an asterisk symbol (*) for each phosphosite (*t*-test two-stage step-up Benjamini, Krieger and Yekutieli; significant values defined as *p* < 0.05). **(B)** Evolutionarily conserved phosphorylation sites that significantly change in response to cLTD, from chicken to Human (Note that Human T241 refers to S241 in mouse). **(C)** Phosphosites from current literature are shown. Sites described in this study are further categorized into phospholevels upregulated (red), downregulated (blue) or unregulated (black) by cLTD. Please note the remaining grey bars represent described sites in literature not detected in the present study.

To investigate the phospho-regulation of DBN in response to cLTD, we performed TMT-based phosphoproteomic analysis on lysates from NMDA-treated and untreated cortical neurons. Across the study, 14 DBN phosphorylation sites were identified. Of these, 11 matched previously reported sites, while three novel sites—S335, S339, and S341—were detected within the adult-specific insert (ASI) of DBN A (Table 1). Most sites were confidently localized, with phospho-site probabilities ≥0.75. Quantitative analysis revealed that 9 phospho-sites (T241, S274, S339, S341, S342, T377, S385, S388, and S647) showed significant decreases in phosphorylation following NMDA treatment, whereas two sites (S142 and T392) displayed increased phosphorylation (Figure 3A). The remaining three sites (S141, S335 and S383) did not exhibit significant regulation in response to cLTD.

**TABLE 1:** DBN phosphorylation sites identified during phosphoproteomic analysis and respective confidence values.

Position (human)	Position (mouse)	P-probability	Classification
S141	S141	0.932381	Robust
S142	S142	0.998566	Robust
T241	S241	1	Robust
S274	S274	0.999637	Robust
S335	S337	0.850278	Robust
S339	S341	0.881376	Robust
S341	S343	0.613765	Medium
S342	S344	0.999284	Robust
T377	T379	0.999929	Robust
S383	S385	0.952643	Robust
S385	S387	0.69861	Medium
S388	S390	0.65741	Medium
T392	T394	0.932571	Robust
S647	S658	0.986376	Robust

Regardless of their phosphorylation dynamics, all identified sites are evolutionarily conserved from chicken (*Gallus gallus*) to Human (*Homo sapiens sapiens*) (Figure 3B). Analysis of the spatial distribution of the phosphosites revealed no clear clustering pattern among either the upregulated or downregulated residues along the DBN domains. Two identified sites downregulated in response to NMDA (T241 and S274) are located within the helical actin-binding domain, while three downregulated sites (S339, S341, and S342) are situated within the serine-rich region of the ASI of DBN A. The remaining phosphosites are distributed throughout the protein, with a minor grouping observed just downstream of the ASI region (Figure 3C).

## Discussion

DBN is a key actin-binding protein that stabilizes filamentous actin, particularly within dendritic spines, where it plays a critical role in regulating spine morphogenesis and synaptic plasticity. Its loss has been implicated in age-related memory decline and neurodegenerative diseases such as AD, suggesting that DBN contributes to maintaining synaptic integrity during aging and under pathological conditions. Despite its functional importance, our understanding of how DBN is regulated during synaptic plasticity remains limited. Phosphorylation—one of the most rapid, reversible, and versatile post-translational modifications—is commonly linked to the modulation of protein activity and conformational states. DBN is known to be extensively phosphorylated, with 40 sites identified to date. However, the regulatory significance of most of these sites remains unclear. To address this gap, we performed a comprehensive phosphoproteomic analysis of the neuron-specific DBN A isoform.

Our study reveals that DBN undergoes distinct phosphorylation changes in response to NMDA-induced cLTD in cortical neurons. Strikingly, with the exception of S142 and T392, which were upregulated, the majority of DBN phosphosites were dephosphorylated following cLTD. One of the upregulated sites, S142, is a known target of Cdk5—a kinase frequently implicated in synaptic remodelling and neurodegenerative diseases. Phosphorylation at S142 is known to modulate DBN’s intramolecular conformation (Worth et al., 2013). Its upregulation suggests a shift toward an open conformation, potentially influencing DBN’s interaction with actin filaments and binding partners. Importantly, aberrant Cdk5 activity and dysregulation of DBN have been linked to synaptic dysfunction in AD, suggesting that similar phosphorylation-dependent mechanisms may be disrupted in disease (Pao and Tsai, 2021; Shah and Rossie, 2017). In contrast, S647—phosphorylated by ATM kinase and implicated in protein stability (Kreis et al., 2019)—was significantly downregulated. Given that reduced ATM signalling and increased DBN degradation have both been reported in models of neurodegeneration (Harigaya et al., 1996; Shen et al., 2016; Shim and Lubec, 2002), the decrease in pS647 may reflect a pathway by which synaptic activity triggers controlled DBN turnover. Dysregulation of this process could lead to synaptic destabilization in pathological contexts.

Furthermore, several regulated sites (S339, S341, S342) lie within the ASI of DBN A, indicating isoform-specific modulation during synaptic plasticity. Interestingly Tanabe et al. (2014) have previously identified S342 as a phosphorylation target of Cdk5, tightly regulating neuronal migration during cortical development. Recent studies also suggest that the ASI contributes to tighter actin binding, particularly through residues Y352 and C355 (Srapyan et al., 2025). Although none of the phosphorylation events identified in our study directly overlap with those reported by Srapyan et al. (2025) the presence of a phosphorylation cluster in the same region underscores its potential functional importance. This clustering, particularly within the ASI, may reflect a regulatory hotspot that modulates DBN’s interaction with the actin cytoskeleton or other synaptic components. One hypothesis is that dephosphorylation in the ASI region weakens the unique actin-binding activity of DBN A, thereby promoting the actin depolymerization required for spine shrinkage during LTD. In this way, our findings offer mechanistic insight into how DBN phosphorylation may serve as a regulatory axis linking synaptic activity to structural plasticity and, when impaired, to disease progression.

In addition, T241 and S274—located within the helical domain of DBN’s second actin-binding region—emerge as compelling candidates for the regulation of DBN’s actin-binding activity. Given DBN’s established role in stabilizing F-actin at dendritic spines, phosphorylation at these sites may represent a mechanism to fine-tune its function in response to synaptic signals.

A small cluster of phosphosites is found between the ASI and the proline-rich domain of DBN A, with phosphorylation levels at residues T377, S385, S388 going down and at residue T392 going up. Without further evidence on specific kinases/phosphatase and regulatory stimuli, these sites remain to have their functional outcome elucidated.

Notably, no phosphosites were detected within the ADF and AB1 domains, or in the proximal regions of the P and H domains under both steady-state and NMDA-induced cLTD conditions, suggesting that sites within these domains may not be regulated in neurons.

It is particularly intriguing to consider how phosphorylation and dephosphorylation events might impact DBN’s structural conformation, stability, or turnover. In neurodegenerative diseases such as AD, DBN levels are often reduced, mislocalised, or degraded—coinciding with synaptic weakening and spine loss (Harigaya et al., 1996; Ishizuka and Hanamura, 2017; Shim and Lubec, 2002). If phosphorylation at specific sites governs DBN’s protective role at the synapse, dysregulation of these modifications could contribute to synaptic vulnerability in aging and disease. Our findings provide a framework for understanding how activity-dependent DBN phosphorylation may support synaptic resilience, and highlight the need for further functional studies to determine the precise impact of these modifications on neuronal health.

Calpains are calcium-dependent cysteine proteases involved in numerous physiological processes, including cytoskeletal remodelling, synaptic plasticity, and protein turnover. However, their dysregulation has been strongly linked to neurodegenerative conditions such as AD, Parkinson’s disease, and Huntington’s disease, where excessive calpain activity contributes to synaptic degradation and neuronal death (Metwally et al., 2023). Calpain is activated in response to elevated intracellular calcium levels—commonly seen during excitotoxicity—and cleaves a range of substrates critical for maintaining neuronal architecture. In our study, we observed that NMDA-induced cLTD in cortical neurons leads to DBN dephosphorylation and cleavage by calpain, likely triggered by NMDA-dependent calcium influx. Previous data showed that dephosphorylation at S647 destabilises DBN, suggesting a mechanism in which calcium-dependant phosphatases, e.g., calcineurin, could render the protein more susceptible to cleavage by calpain (Kreis et al., 2013). Another possible mechanism could involve the PTEN phosphatase, known to dephosphorylate DBN at S647 and to be recruited to the synapse in response to LTD (Jurado et al., 2010; Kreis et al., 2013). The resulting cleavage by calpain yielded a prominent N-terminal fragment of approximately 45–50 kDa. Given that this region contains critical domains for actin binding and conformational regulation, cleavage in this region could significantly alter DBN’s interaction with the cytoskeleton or other synaptic proteins. In fact it is plausible that lacking the C-terminal region, this fragment may act as a dominant negative. Interestingly, DBN has previously been identified as a calpain substrate under excitotoxic conditions (Chimura et al., 2015). Although the excitotoxicity protocol (NMDA 30 μM for 2.5 h) is very different from our cLTD protocol (5 min pulse of 50 μM NMDA), they also identified similar sized fragments. When comparing these two studies, our findings suggest that calpain-mediated cleavage of DBN may not be limited to pathological states but may also play a role in activity-dependent synaptic remodelling. Whether the cleavage of DBN leads to a loss of DBN or whether the remaining fragments still retain some form of activity remains to be elucidated.

In neurodegenerative diseases such as AD, prolonged or uncontrolled calpain activation—driven by chronic calcium dysregulation—could lead to excessive DBN degradation. This would compromise actin stability within dendritic spines, ultimately contributing to synapse loss and cognitive decline. In this context, our acute *in vitro* findings suggest that the physiological calpain-dependent cleavage of DBN observed during cLTD may represent a finely tuned mechanism for remodelling the spine cytoskeleton, which becomes pathologically amplified under disease conditions.

Together, our findings highlight calpain as a critical regulator of DBN stability and suggest a dual role for this protease in both normal synaptic plasticity and disease-related synaptic dysfunction. Future studies aimed at mapping the exact cleavage site and functional consequences of the DBN fragment will be essential to better understand how DBN processing contributes to synaptic health and pathology.

## Data Availability

The original contributions presented in the study are publicly available in the ProteomeXchange Consortium via the PRIDE partner repository, with dataset identifier PXD069538 (https://www.ebi.ac.uk/pride/archive/projects/PXD069538).

## Supplementary material

Supplementary Figure 1 Validation of the induction of cLTD by analysis of GluR1 phospholevels. To access the efficiency of our cLTD protocol we analyzed the phosphorylation status of GluR1 using our phosphoproteomic data. We show that following NMDA mediated cLTD, GluR1 phosphorylation sites S863 and T858 but not S849 were decreased in accordance with previous literature (Delgado et al., 2007; Hsin et al., 2010; Lee et al., 1998). Heatmap shows the average ratio of occupation at each site by phosphorylation for each GluR1 phosphosite identified during proteomics in four individual neuronal cultures. Values represented are the log_2_ intensity values normalized to the median z-score within each sample. Increasing red or blue values represent higher levels of phosphorylation or lower levels of phosphorylation, respectively. Significantly altered phosphorylation levels are indicated in bold together with an asterisk symbol (*) for each phosphosite (*t*-test two-stage step-up Benjamini, Krieger and Yekutieli; significant values defined as *p* < 0.05).

Supplementary Figure 2 Calpain-mediated DBN cleavage generates N-terminal fragments. **(A)** Full length-DBN levels are decreased between untreated (C) and NMDA-treated (N) neuronal lysates. Calpain-mediated cleavage of DBN produces N-terminal and middle domains fragments with several molecular weights, but not C-terminal fragments - as seen by the use of three DBN antibodies with different epitopes. The most prominent fragment around 45–50 kDa is again highlighted (*). Molecular weight protein ladder is in kilodaltons. The C-term antibody (non-pS647-DBN) was developed in Kreis et al. (2013). **(B)** Calpain cleavage prediction on mouse DBN A, with single dots representing each potential cleavage site with a score higher than 0.65. DBN domains are superimposed to facilitate interpretation. *In silico* prediction made with CalCleaveMKL.

## References

[B1] Biou V. Brinkhaus H. Malenka R. Matus A. (2008). Interactions between drebrin and Ras regulate dendritic spine plasticity. *Eur. J. Neurosci.* 27 2847–2859. 10.1111/j.1460-9568.2008.06269.x 18588530

[B2] Bosch M. Castro J. Saneyoshi T. Matsuno H. Sur M. Hayashi Y. (2014). Structural and molecular remodeling of dendritic spine substructures during long-term potentiation. *Neuron* 82 444–459. 10.1016/j.neuron.2014.03.021 24742465 PMC4281348

[B3] Chimura T. Launey T. Yoshida N. (2015). Calpain-mediated degradation of drebrin by excitotoxicity in vitro and in vivo. *PLoS One* 10:e0125119. 10.1371/journal.pone.0125119 25905636 PMC4408054

[B4] Citri A. Malenka R. (2008). Synaptic plasticity: Multiple forms, functions, and mechanisms. *Neuropsychopharmacology* 33 18–41. 10.1038/sj.npp.1301559 17728696

[B5] Counts S. He B. Nadeem M. Wuu J. Scheff S. Mufson E. (2012). Hippocampal drebrin loss in mild cognitive impairment. *Neurodegener. Dis.* 10 216–219. 10.1159/000333122 22310934 PMC3363353

[B6] Delgado J. Coba M. Anderson C. Thompson K. Gray E. Heusner C. (2007). NMDA receptor activation dephosphorylates AMPA receptor glutamate receptor 1 subunits at threonine 840. *J. Neurosci.* 27 13210–13221. 10.1523/JNEUROSCI.3056-07.2007 18045915 PMC2851143

[B7] Geraldo S. Khanzada U. Parsons M. Chilton J. Gordon-Weeks P. (2008). Targeting of the F-actin-binding protein drebrin by the microtubule plus-tip protein EB3 is required for neuritogenesis. *Nat. Cell. Biol.* 10 1181–1189. 10.1038/ncb1778 18806788

[B8] Harigaya Y. Shoji M. Shirao T. Hirai S. (1996). Disappearance of actin-binding protein, drebrin, from hippocampal synapses in Alzheimer’s disease. *J. Neurosci. Res.* 43 87–92. 10.1002/jnr.490430111 8838578

[B9] Hatanpää K. Isaacs K. Shirao T. Brady D. Rapoport S. (1999). Loss of proteins regulating synaptic plasticity in normal aging of the human brain and in Alzheimer disease. *J Neuropathol. Exp. Neurol.* 58 637–643. 10.1097/00005072-199906000-00008 10374754

[B10] Hsin H. Kim M. Wang C. Sheng M. (2010). Proline-rich tyrosine kinase 2 regulates hippocampal long-term depression. *J. Neurosci.* 30 11983–11993. 10.1523/JNEUROSCI.1029-10.2010 20826662 PMC4122232

[B11] Hughes C. Moggridge S. Müller T. Sorensen P. Morin G. Krijgsveld J. (2019). Single-pot, solid-phase-enhanced sample preparation for proteomics experiments. *Nat. Protoc.* 14 68–85. 10.1038/s41596-018-0082-x 30464214

[B12] Ishizuka Y. Hanamura K. (2017). Drebrin in Alzheimer’s disease. *Adv. Exp. Med. Biol.* 1006 203–223. 10.1007/978-4-431-56550-5_12 28865022

[B13] Ivanov A. Esclapez M. Pellegrino C. Shirao T. Ferhat L. (2009). Drebrin A regulates dendritic spine plasticity and synaptic function in mature cultured hippocampal neurons. *J. Cell. Sci.* 122 524–534. 10.1242/jcs.033464 19174472

[B14] Jurado S. Benoist M. Lario A. Knafo S. Petrok C. Esteban J. A. (2010). PTEN is recruited to the postsynaptic terminal for NMDA receptor-dependent long-term depression. *EMBO J.* 29 2827–2840. 10.1038/emboj.2010.160 20628354 PMC2924645

[B15] Kallergi E. Daskalaki A. Kolaxi A. Camus C. Ioannou E. Mercaldo V. (2022). Dendritic autophagy degrades postsynaptic proteins and is required for long-term synaptic depression in mice. *Nat. Commun.* 13 1–23. 10.1038/s41467-022-28301-z 35115539 PMC8814153

[B16] Kapitein L. Yau K. Gouveia S. van der Zwan W. Wulf P. Keijzer N. (2011). NMDA receptor activation suppresses microtubule growth and spine entry. *J. Neurosci.* 31 8194–8209. 10.1523/JNEUROSCI.6215-10.2011 21632941 PMC6622869

[B17] Keon B. Jedrzejewski P. Paul D. Goodenough D. (2000). Isoform specific expression of the neuronal F-actin binding protein, drebrin, in specialized cells of stomach and kidney epithelia. *J. Cell. Sci.* 113 325–336. 10.1242/jcs.113.2.325 10633083

[B18] Klemz A. Kreis P. Eickholt B. Gerevich Z. (2021). The actin binding protein drebrin helps to protect against the development of seizure-like events in the entorhinal cortex. *Sci. Rep.* 11:8662. 10.1038/s41598-021-87967-5 33883605 PMC8060314

[B19] Kreis P. Gallrein C. Rojas-Puente E. Mack T. Kroon C. Dinkel V. (2019). ATM phosphorylation of the actin-binding protein drebrin controls oxidation stress-resistance in mammalian neurons and C. elegans. *Nat. Commun.* 10:486. 10.1038/s41467-019-08420-w 30700723 PMC6353951

[B20] Kreis P. Hendricusdottir R. Kay L. Papageorgiou I. van Diepen M. Mack T. (2013). Phosphorylation of the actin binding protein Drebrin at S647 is regulated by neuronal activity and PTEN. *PLoS One* 8:e71957. 10.1371/journal.pone.0071957 23940795 PMC3733845

[B21] Lee H. Kameyama K. Huganir R. Bear M. F. (1998). NMDA induces long-term synaptic depression and dephosphorylation of the GluR1 subunit of AMPA receptors in hippocampus. *Neuron* 21 1151–1162. 10.1016/s0896-6273(00)80632-7 9856470

[B22] Lüscher C. Malenka R. C. (2012). NMDA receptor-dependent long-term potentiation and long-term depression (LTP/LTD). *Cold Spring Harb. Perspect. Biol.* 4:a005710. 10.1101/cshperspect.a005710 22510460 PMC3367554

[B23] Martínez-Mármol R. Barneda-Zahonero B. Soto D. Andrés R. Coccia E. Gasull X. (2016). FAIM-L regulation of XIAP degradation modulates synaptic long-term depression and axon degeneration. *Sci. Rep.* 6:35775. 10.1038/srep35775 27767058 PMC5073314

[B24] Metwally E. Al-Abbadi H. Hussain T. Murtaza G. Abdellatif A. Ahmed M. (2023). Calpain signaling: From biology to therapeutic opportunities in neurodegenerative disorders. *Front. Vet. Sci.* 10:1235163. 10.3389/fvets.2023.1235163 37732142 PMC10507866

[B25] Mikati M. Grintsevich E. Reisler E. (2013). Drebrin-induced stabilization of actin filaments. *J. Biol. Chem.* 288 19926–19938. 10.1074/jbc.M113.472647 23696644 PMC3707693

[B26] Mizui T. Takahashi H. Sekino Y. Shirao T. (2005). Overexpression of drebrin A in immature neurons induces the accumulation of F-actin and PSD-95 into dendritic filopodia, and the formation of large abnormal protrusions. *Mol. Cell. Neurosci.* 30 149–157. 10.1016/j.mcn.2005.06.008 16054392

[B27] Pao P. Tsai L. (2021). Three decades of Cdk5. *J. Biomed. Sci.* 28 1–17. 10.1186/s12929-021-00774-y 34814918 PMC8609871

[B28] Schrötter S. Leondaritis G. Eickholt B. (2016). Capillary isoelectric focusing of Akt isoforms identifies highly dynamic phosphorylation in neuronal cells and brain tissue. *J. Biol. Chem.* 291 10239–10251. 10.1074/jbc.M115.700138 26945062 PMC4858973

[B29] Sekino Y. Tanaka S. Hanamura K. Yamazaki H. Sasagawa Y. Xue Y. (2006). Activation of N-methyl-D-aspartate receptor induces a shift of drebrin distribution: Disappearance from dendritic spines and appearance in dendritic shafts. *Mol. Cell. Neurosci.* 31 493–504. 10.1016/j.mcn.2005.11.003 16368245

[B30] Shah K. Rossie S. (2017). Tale of the good and the bad Cdk5: Remodeling of the actin cytoskeleton in the brain. *Mol. Neurobiol.* 55 3426–3438. 10.1007/s12035-017-0525-3 28502042 PMC6370010

[B31] Sharma S. Grintsevich E. Phillips M. Reisler E. Gimzewski J. (2011). Atomic force microscopy reveals drebrin induced remodeling of f-actin with subnanometer resolution. *Nano Lett.* 11 825–827. 10.1021/nl104159v 21175132 PMC3670797

[B32] Shen X. Chen J. Li J. Kofler J. Herrup K. (2016). Neurons in vulnerable regions of the Alzheimer’s disease brain display reduced ATM signaling. *eNeuro* 3:ENEURO.0124–15.2016. 10.1523/ENEURO.0124-15.2016 27022623 PMC4770009

[B33] Shim K. Lubec G. (2002). Drebrin, a dendritic spine protein, is manifold decreased in brains of patients with Alzheimer’s disease and Down syndrome. *Neurosci. Lett.* 324 209–212. 10.1016/s0304-3940(02)00210-0 12009525

[B34] Shirao T. Sekino Y. (2017). *Drebrin: From structure and function to physiological and pathological roles.* Tokyo: Springer, 401.

[B35] Shirao T. Hanamura K. Koganezawa N. Ishizuka Y. Yamazaki H. Sekino Y. (2017). The role of drebrin in neurons. *J. Neurochem.* 141 819–834. 10.1111/jnc.13988 28199019

[B36] Sonego M. Oberoi M. Stoddart J. Gajendra S. Hendricusdottir R. Oozeer F. (2025). Drebrin regulates neuroblast migration in the postnatal mammalian brain. *PLoS One* 10:e0126478. 10.1371/journal.pone.0126478 25945928 PMC4422745

[B37] Srapyan S. Mkrtchyan M. Berlemont R. Grintsevich E. (2025). Functional differences between neuronal and non-neuronal isoforms of drebrin. *J. Mol. Biol.* 437:169015. 10.1016/j.jmb.2025.169015 39971265 PMC12974149

[B38] Tanabe K. Yamazaki H. Inaguma Y. Asada A. Kimura T. Takahashi J. (2014). Phosphorylation of drebrin by cyclin-dependent kinase 5 and its role in neuronal migration. *PLoS One* 9:e92291. 10.1371/journal.pone.0092291 24637538 PMC3956921

[B39] Varga S. Kaasen J. Gáspári Z. Péterfia B. Mulder F. (2025). Resonance assignment of the intrinsically disordered actin-binding region of Drebrin. *Biomol. NMR Assign.* 19 221–225. 10.1007/s12104-025-10239-0 40515930 PMC12513958

[B40] Willmes C. Mack T. Ledderose J. Schmitz D. Wozny C. Eickholt B. (2017). Investigation of hippocampal synaptic transmission and plasticity in mice deficient in the actin-binding protein Drebrin. *Sci. Rep.* 7:42652. 10.1038/srep42652 28198431 PMC5309812

[B41] Worth D. Daly C. Geraldo S. Oozeer F. Gordon-Weeks P. (2013). Drebrin contains a cryptic F-actin-bundling activity regulated by Cdk5 phosphorylation. *J. Cell. Biol.* 202 793–806. 10.1083/jcb.201303005 23979715 PMC3760615

[B42] Yamazaki H. Shirao T. (2017). Homer, spikar, and other drebrin-binding proteins in the brain. *Adv. Exp. Med. Biol.* 1006 249–268. 10.1007/978-4-431-56550-5_14 28865024

