# Supplementary figures and images for: Phosphoprofile reorganization of the actin binding protein Drebrin during long term depression
Source: Front Mol Neurosci. 2025 Nov 5;18:1697642. doi: 10.3389/fnmol.2025.1697642 (PMC12627054; doi:10.3389/fnmol.2025.1697642)

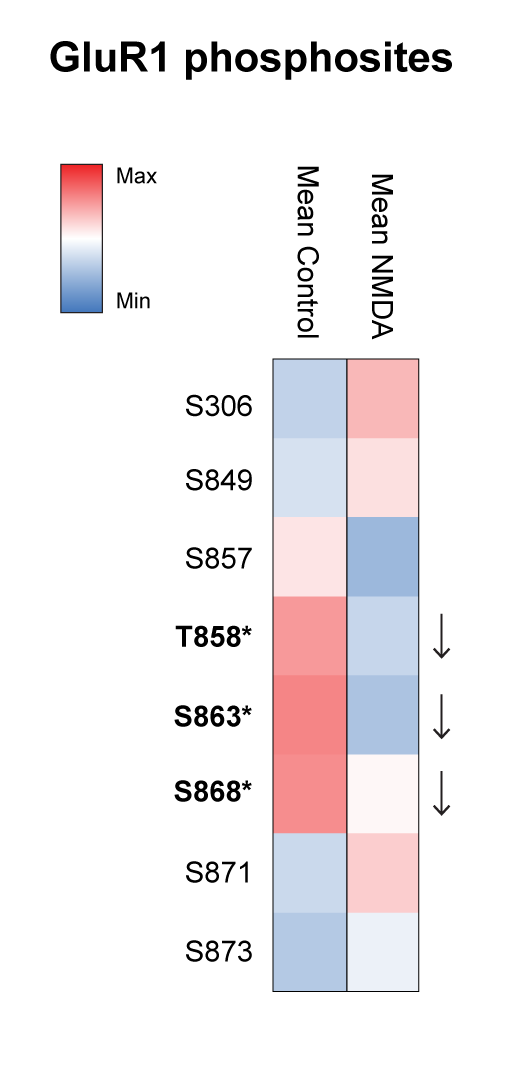

Supplement: Supplementary Figure 1 — Validation of the induction of cLTD by analysis of GluR1 phospholevels. To access the efficiency of our cLTD protocol we analyzed the phosphorylation status of GluR1 using our phosphoproteomic data. We show that following NMDA mediated cLTD, GluR1 phosphorylation sites S863 and T858 but not S849 were decreased in accordance with previous literature (Delgado et al., 2007; Hsin et al., 2010; Lee et al., 1998). Heatmap shows the average ratio of occupation at each site by phosphorylation for each GluR1 phosphosite identified during proteomics in four individual neuronal cultures. Values represented are the log2 intensity values normalized to the median z-score within each sample. Increasing red or blue values represent higher levels of phosphorylation or lower levels of phosphorylation, respectively. Significantly altered phosphorylation levels are indicated in bold together with an asterisk symbol (*) for each phosphosite (t-test two-stage step-up Benjamini, Krieger and Yekutieli; significant values defined as p < 0.05). [file Image_1.tif]

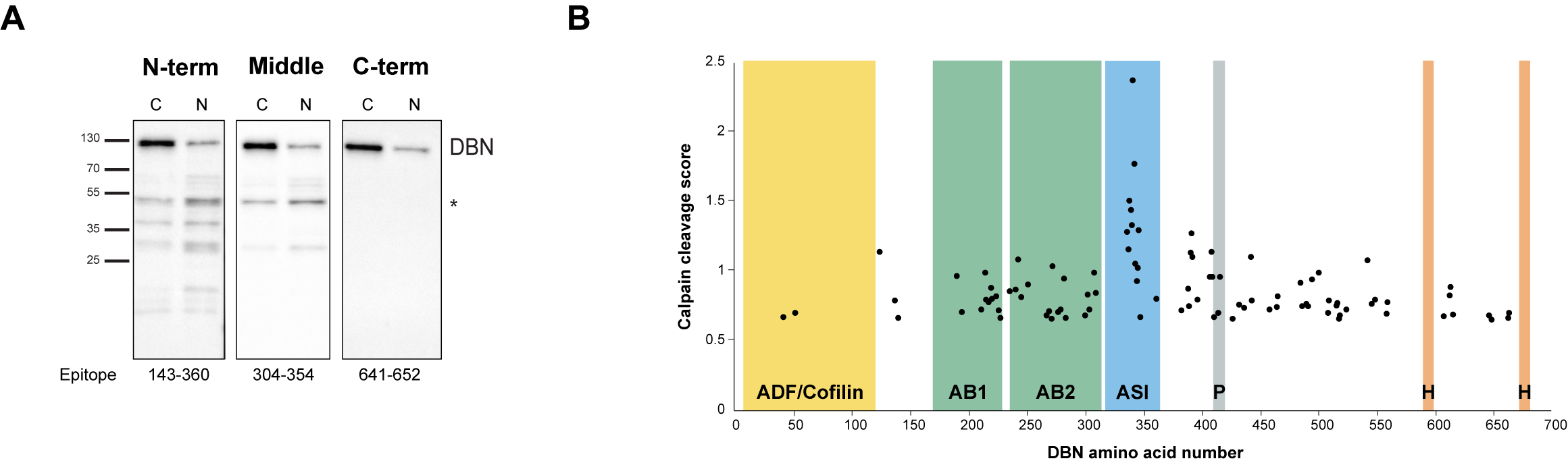

Supplement: Supplementary Figure 2 — Calpain-mediated DBN cleavage generates N-terminal fragments. (A) Full length-DBN levels are decreased between untreated (C) and NMDA-treated (N) neuronal lysates. Calpain-mediated cleavage of DBN produces N-terminal and middle domains fragments with several molecular weights, but not C-terminal fragments - as seen by the use of three DBN antibodies with different epitopes. The most prominent fragment around 45–50 kDa is again highlighted (*). Molecular weight protein ladder is in kilodaltons. The C-term antibody (non-pS647-DBN) was developed in Kreis et al. (2013). (B) Calpain cleavage prediction on mouse DBN A, with single dots representing each potential cleavage site with a score higher than 0.65. DBN domains are superimposed to facilitate interpretation. In silico prediction made with CalCleaveMKL. [file Image_2.tif]
